# A mobile app implementing the international classification of functioning, disability and health rehabilitation set

**DOI:** 10.1186/s12911-020-1019-1

**Published:** 2020-01-28

**Authors:** Malan Zhang, Jiani Yu, Wei Shen, Yun Zhang, Yun Xiang, Xinting Zhang, Ziling Lin, Tiebin Yan

**Affiliations:** 1 Department of Rehabilitation, Sun Yat-sen Memorial Hospital, Sun Yat-sen University, Guangzhou, 510120 China; 2 Hexian Memorial Affiliated Hospital of Southern Medical University, Guangzhou, 511400 China; 3 GuangDong 999Brain Hospital, Guangzhou, China; 4 The Fifth Hospital of Xiamen, Xiamen, China; 5 Shenzhen Nanshan People’s Hospital, Shenzhen, China; 6 Clifford Hospital, Guangzhou, China; 7 The Fifth Affiliated Hospital, Sun Yat-sen University, Zhuhai, China; 8 Engineering Technology Research Center for Rehabilitation and Elderly Care, Sun Yat-sen University, Guangzhou, China

**Keywords:** Assessment, International classification of functioning disability and health, Mobile applications, Rehabilitation

## Abstract

**Background:**

The Chinese assessment standards of the International Classification of Functioning, Disability and Health Rehabilitation Set is available now. It is coming to be used as a basic functional evaluation tool in China. With data accumulating, a mobile application is needed to eliminate the extra cost of data entry, storage, and graphical presentation of trends. This study aimed to design, develop and test a mobile app based on the International Classification of Functioning, Disability and Health Rehabilitation Set Rehabilitation Set.

**Methods:**

The study had three phases. The first involved specifying the functional requirements of the app. Then an app was designed and refined to meet those requirements. In a pilot test, the app was used by rehabilitation professionals in clinical practice and their comments were collected for its further modification in one-on-one interviews.

**Results:**

The app met the initial requirements, and the pilot study showed it worked as designed. The pilot study also showed that the app is user-friendly and convenient to use in rehabilitation practice. Some feedback was given to improve the app.

**Conclusion:**

An Android mobile app implementing the International Classification of Functioning, Disability and Health Rehabilitation Set was successfully developed.

## Background

In the twenty-first century the global demand for medical rehabilitation continues to increase, especially in low- and middle-income countries [1]. Functioning is the key indicator of successful rehabilitation [2, 3], so the functional evaluation tools shared by professionals are very important for rehabilitation practice. The International Classification of Functioning and Health (ICF) endorsed by the World Health Organization (WHO) in 2001 is considered the best unified framework for documenting and reporting functioning based on biological, psychological, and social perspectives [4–7].The ICF conceives of functioning as a dynamic interaction between a person’s health, environmental factors and other personal factors. It has been recommended as a reference system for comparative evaluation and standardized reporting of rehabilitation interventions and their outcomes [8]. To facilitate the use of ICF, an International Classification of Functioning, Disability and Health Rehabilitation Set (ICF-RS) has been developed following a scientific process [9]. It can be used in the context of rehabilitation and disability to describe varying levels of functioning across different clinical populations and along the continuum of care [10]. Due to that the ICF-RS is only a list of 30 categories and lack of detailed operational items, a Chinese version of the assessment standards has been developed based on the established ICF linking rules [11, 12].

The Chinese assessment standards of the ICF-RS have been used across China, but data accumulation has led to some problems. For example, archiving the paper version takes up much storage space, easily leading to data being lost. There is also the not-insignificant cost of data entry. And the paper records can be difficult to share and compare between different institutions. With the rapid development of mobile health technology, mobile applications (apps) may be an effective tool for overcoming such difficulties. A mobile app could facilitate more frequent assessment, allow more objective data collection, save storage space, prevent data loss, and ensure high-speed data delivery [13–15]. It can be used flexibly even without Internet access, making health information available without cite restriction [16]. Mobile health apps have been shown to improve efficiency and value while lowering costs [17]. At least 325,000 health, fitness and medical apps are now available in major app stores. About 78,000 new ones became available in 2017 alone. In that year there were 3.7 billion downloads of mobile health applications, an increase of 16% over 2016 [18]. Several mobile apps for the ICF have been reported [19–21], but none is available yet for the ICF-RS.

The objective of this study, therefore, was to develop a mobile app based on the ICF-RS, test its utility and collect feedback for its further modification. The goal was an app suitable for frequently recording patients’ functional levels which could display functional changes graphically. It was designed to be suitable for tracking patients with different diseases in different medical institutions. At the same time, individual rehabilitation intervention plans can be made on the app based on the evaluation results. The app represents a real-world example of the application and promotion of the ICF-RS. The manuscript is organized as follows. The Methods section describes design and development steps of the app, the main technology used, and the setup of function modules. The Results section describes the developing process of the app; the results of the pilot test and the measures taken based on the feedback are also reported. The Discussion section describes the significance of app development and limitations of the study, followed by the conclusion.

## Methods

The app was developed collaboratively by the Sun Yat-sen Memorial Hospital, Sun Yat-sen University and the Yunrun Big Data Limited Company. A core development group was set up, consisting of a senior professor and a postdoctoral fellow from the Sun Yat-sen Memorial Hospital, a product manager, a prototype designer, and a software development engineer from the company.

### Theoretical foundation

The mobile app used the ICF domains (Table 1) as its theoretical foundation and the ICF-RS as its framework. The ICF-RS has 30 categories (Table 2) selected from the ICF domains to describe functional status during rehabilitation from the acute to the chronic stage [9]. There are 9 categories for body function, 14 categories for activity, and 7 categories for participation. The Chinese assessment standards of the ICF-RS are available on the app (see in Additional file 1: Table S1). Ratings are evaluated through interviews and clinical examination. A qualifier ranging from 0 to 4 is used to describe the severity of the problem. No problem is graded 0; grade 1 indicates a mild problem; grade 2 indicates a moderate problem; grade 3 indicates a severe problem; and grade 4 indicates complete lack of function. There is also a grade 8 which indicates insufficient information to describe the severity of the problem, while a grade 9 indicates inapplicability for a particular patient [6].

**Table 1:** The domains of the ICF categories

Body structures	Body functions	Activities and Participation	Environmental factors
Structures of the nervous system	Mental functions	Learning and applying knowledge	Supplies and technology
The eyes, ears and related structures	Sensory functions and pain	General tasks and demands	Changes in the natural environment and perceptions of the environment
Structures involved in voice and speech	Voice and speech functions	Communication	Support and interrelationships
Structures of the cardiovascular, immunological, and respiratory systems	Functions of the cardiovascular, haematological, immunological and respiratory system	Mobility	Attitude
Structures related to the digestive, metabolic, and endocrine systems	Functions of the digestive, metabolic and endocrine system	Self-care	Service system and policy
Structures related to the genitourinary and reproductive systems	Genitourinary and reproductive function	Domestic life	
Structures related to movement	Neuromusculoskeletal and movement related functions	Interpersonal interactions and relationships	
Skin and related structures	Neuromusculoskeletal	Major life areas	
	Functions of the skin and related structures	Community, social and civic life

**Table 2:** List of the ICF-RS with 30 categories

Body functions	Activities	Participation
b130 Energy and drive functions	d240 Handling stress and other psychological demands	d230 Carrying out daily routine
b134 Sleep functions	d410 Changing basic body position	d470 Using transportation
b152 Emotional functions	d415 Maintaining a body position	d660 Assisting others
b280 Sensation of pain	d420 Transferring oneself	d710 Basic interpersonal interactions
b455 Exercise tolerance functions	d450/d465 Walking/Moving around using equipment	d770 Intimate relationships
b620 Urination functions	d455 Moving around	d850 Remunerative employment
b640 Sexual functions	d510 Washing oneself	d920 Recreation and leisure
b710 Mobility of joint functions	d520 Caring for body parts	
b730 Muscle power functions	d530 Toileting	
	d540 Dressing	
	d550 Eating	
	d570 Looking after one’s health	
	d640 Doing housework	

In this study the Abbreviated Mental Test (AMT) was used to screen patients for inclusion (see in Additional file 2). It is a 10-item scale for cognitive functioning [22]. Its items are scored as right or wrong with one point for each right answer, so the maximum score is 10 [23].

### Study design

The Design Science Research Methodology [24] was applied in this study, which demonstrated its ability to study the connection between research and professional practices by designing, implementing and evaluating artifacts for a specific need. The process was inspired by Peffers’ s three phases: problem identification, solution design, and evaluation [25]. The evaluation in this case involved a pilot study.

### Problem identification

The development work first focused on discussion and understanding the requirements for a useful app. The team’s senior professor and postdoctoral fellow discussed with the corporate team members the basic functionality to be achieved. The postdoctoral fellow then worked with the firm’s technicians to specify the detailed requirements (Table 3).

**Table 3:** Functional requirements for the mobile app

Requirement	Description
User management	The app can be used across different institutions.
The implementation of ICF-RS	Two evaluation versions of ICF-RS and online learning of the app can be realized.
Rehabilitation management	The app can record the whole rehabilitation process of evaluation, goal setting, intervention, and re-evaluation.
Data management	The app can realize data query, export, and presentation of trend graphs.
Data security	The app must have guaranteed data security.

### Solution design

The next phase involved the prototype design, user interface design, and app development. The designer provided mockups and interface design for the app. Content priority, reasonable layout, ease of operation, and ease of learning were maintained as key design criteria. The software development engineer and his team wrote the code and did the initial testing of the app’s functioning, performance and safety. The postdoctoral fellow and the technicians discussed the details of the app’s latest version once a week. A chat group was established to discuss solutions for the many problems encountered during the development process.

A few nonfunctional requisites were also considered important. (1) Usability requirements: It was required to be easy to understand, learn and to use. (2) Safety requirement: Identity authentication, access control, and patient privacy were also important design criteria. (3) External interface: The app was designed to be connected to a medical records system. (4) Performance requirements: The key performance requirements included the app’ speed of response, result accuracy, and low-runtime resource consumption. (5) Stability: It was also important that the app operate its various functions stably. (6) Extensible and easy to maintain: The design also considered the ability of the app to extend or upgrade in the future. And when failure occurs in actual operation, service must be restored as quickly as possible.

### The technology and architecture

**Fig. 1:**
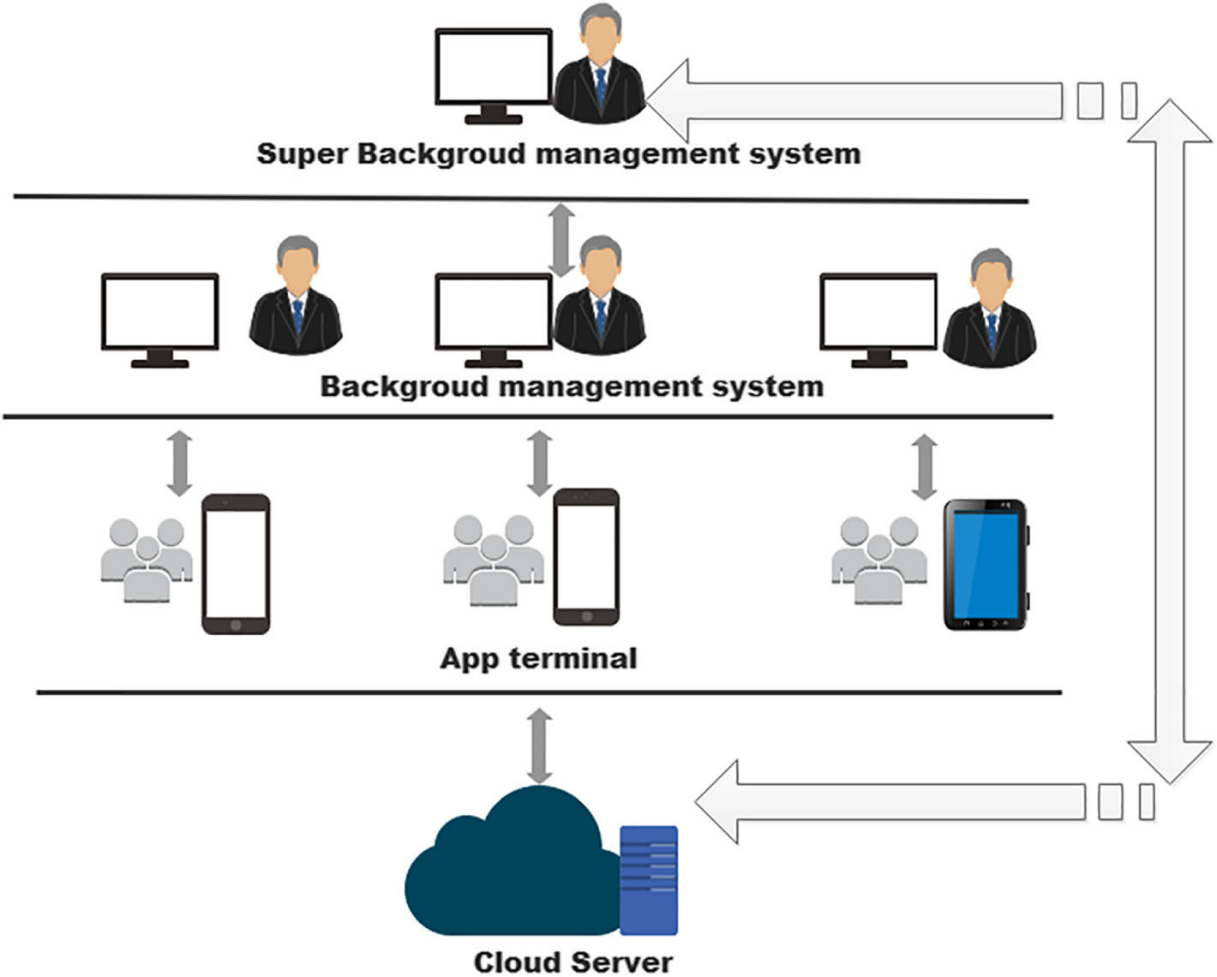
The app’ s archtecture. The app consists of a user interface and a background management system operated by the administrator. Each institution has separate background management system. The server maintains connection with the app terminals and the background management system to realize data interaction and storage

**Fig. 2:**
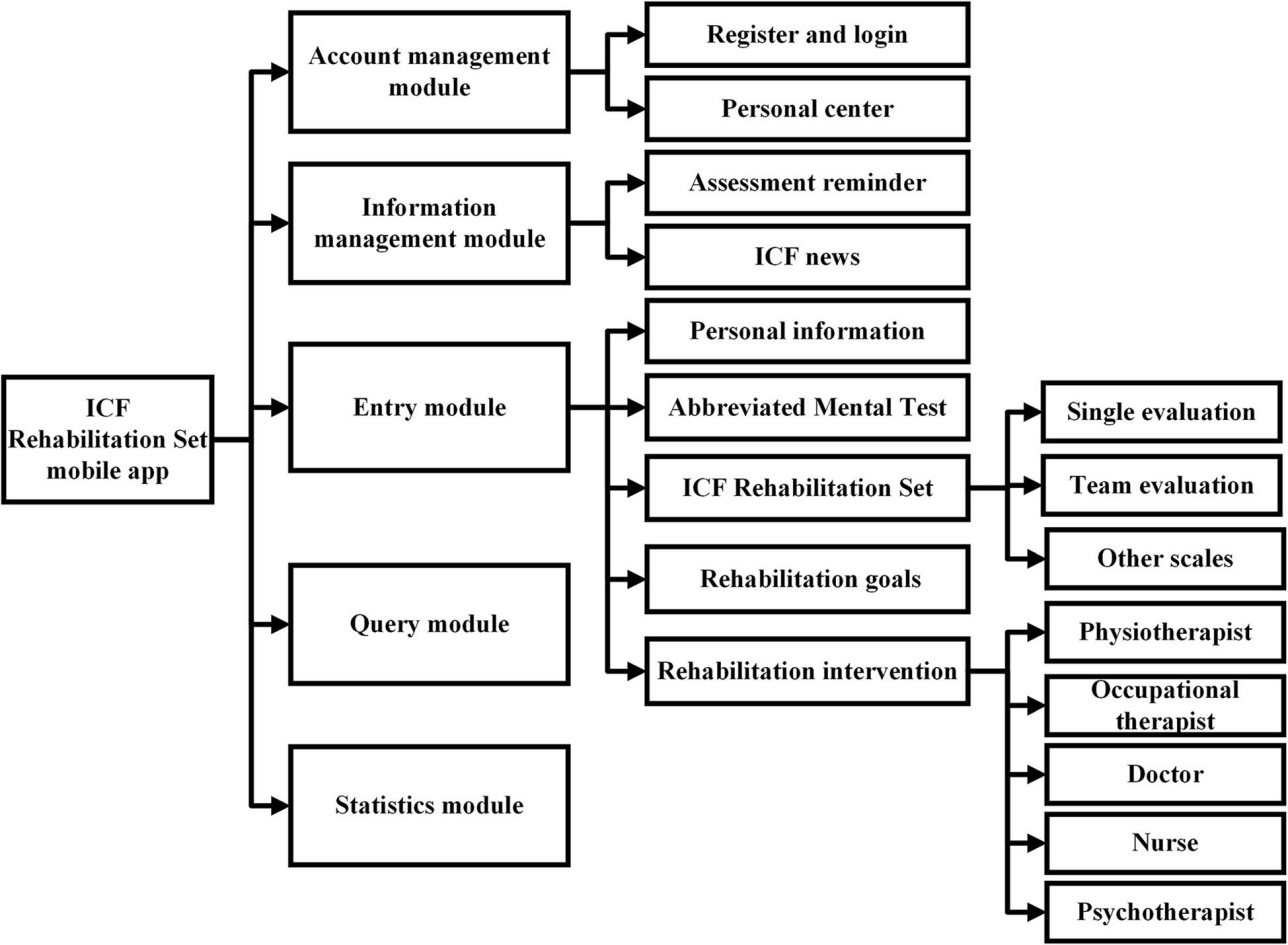
The app’ s fucntional framework. The mobile app includes an account management module, an information management module, an entry module, a query module, and a statistics module

The Java technology was used as programming language. Microsoft structure quest language database technology was used to construct data storage and management database. The app has an interface with which the rehabilitation profession interacts, and a background management system operated by the administrator. A cloud server maintains connection with the app terminal and the background management system. Each institution has separate background management system (Fig. 1). The app was developed for the Android operating system as Android phones are more widely used and more affordable for most people in China. The app terminal was designed to contain an account management module, an information management module, an entry module, a query module, and a statistics module. The entry module was intended to be the most used part. It covered the personal information, the assessment scales, a questionnaire for developing a patient’s rehabilitation goals and a rehabilitation intervention tracker (Fig. 2). The detailed descriptions for the function modules are as follows.

### The account and information management modules

The app was designed to be used in different sorts of rehabilitation institutions, but in China there is still no consensus about data sharing among different rehabilitation institutions. To solve this problem, separate background management accounts were created to ensure data privacy. The scalability was also considered and background accounts can be set up if more hospitals want to use the app. A supervising admin account was set up to monitor the whole dataset. The administrator checks in the background regularly to approve new registrations. When registering, the user needs to specify an institution. The data each user reports can be checked in the app interface and in that institution’s background management system. The information management module also provides next assessment reminders and releases the latest information about the ICF.

### The entry module

The personal information includes both general and disease-related information. After login, the patient information is added by the professional using the app. The AMT should be administered and the scores are recorded. Only patients with AMT scores of six or more can be included in the subsequent formal evaluation. They are invited to sign an electronic informed consent form. Multiple tests involved making an appointment using the app, and the frequency of assessment is decided based on the patient’s situation and is not restricted. Besides the ICF-RS, other scales could be added in the background if necessary, such as the ICF genetic core sets, the Modified Barthel Index, and others. Raters can choose different scales at any time as required and save the results after the evaluation. A patient’s questionnaire was designed to record each patient’s short-term and long-term rehabilitation goals. The goals should include functioning, activity and participation components that the patient hopes to achieve through rehabilitation. The professionals can select relevant categories and record detailed intervention plans combined with the goals for the patient. The evaluation of ICF-RS consists of two main features: two evaluation versions and online learning.

Two evaluation versions of ICF-RS are available on the app. Team assessment can involve doctors, nurses, physiotherapists, occupational therapists, each completing the categories most related to their work. The categories assignments are pre-allocated and bound to specific professionals in the background. Once a professional has created a patient’s record, he or she can choose a doctor, a nurse, a physiotherapist, and/or an occupational therapist to set up an evaluation team. Each professional on the team can see the results of the 30 categories if they were finished on their app.

**Fig. 3:**
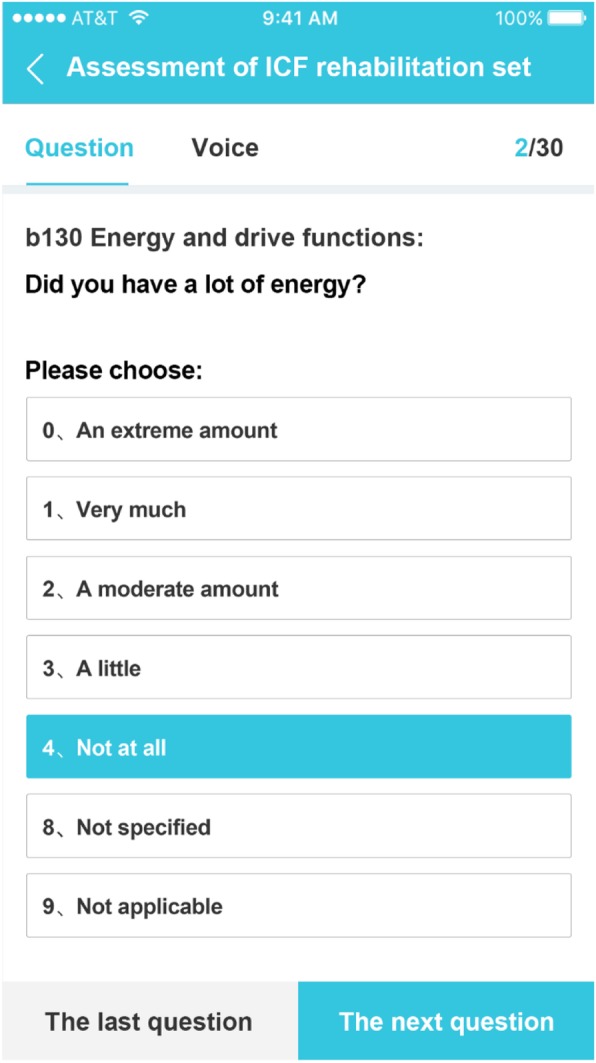
An evaluation page of the ICF-RS category. Each ICF-RS category includes category name, assessment content and outcome options. Both text and voice demonstrations of how to ask the questions are available

The app was also designed to facilitate online learning about ICF assessment. The content and outcome options for each category are presented along with the relevant definitions and descriptions, question demonstrations, and assessment guidance. The whole is set up to help raters in their assessments. The definitions and descriptions of each category in both Chinese and English can be clicked on at any time. The question demonstrations allow for both text and voice presentation (Fig. 3). The assessment guidance includes notes for evaluation, drawings and video demonstrations. Evaluation results are automatically tabulated and can be downloaded after the final answers have been submitted.

### The query and statistics modules

**Fig. 4:**
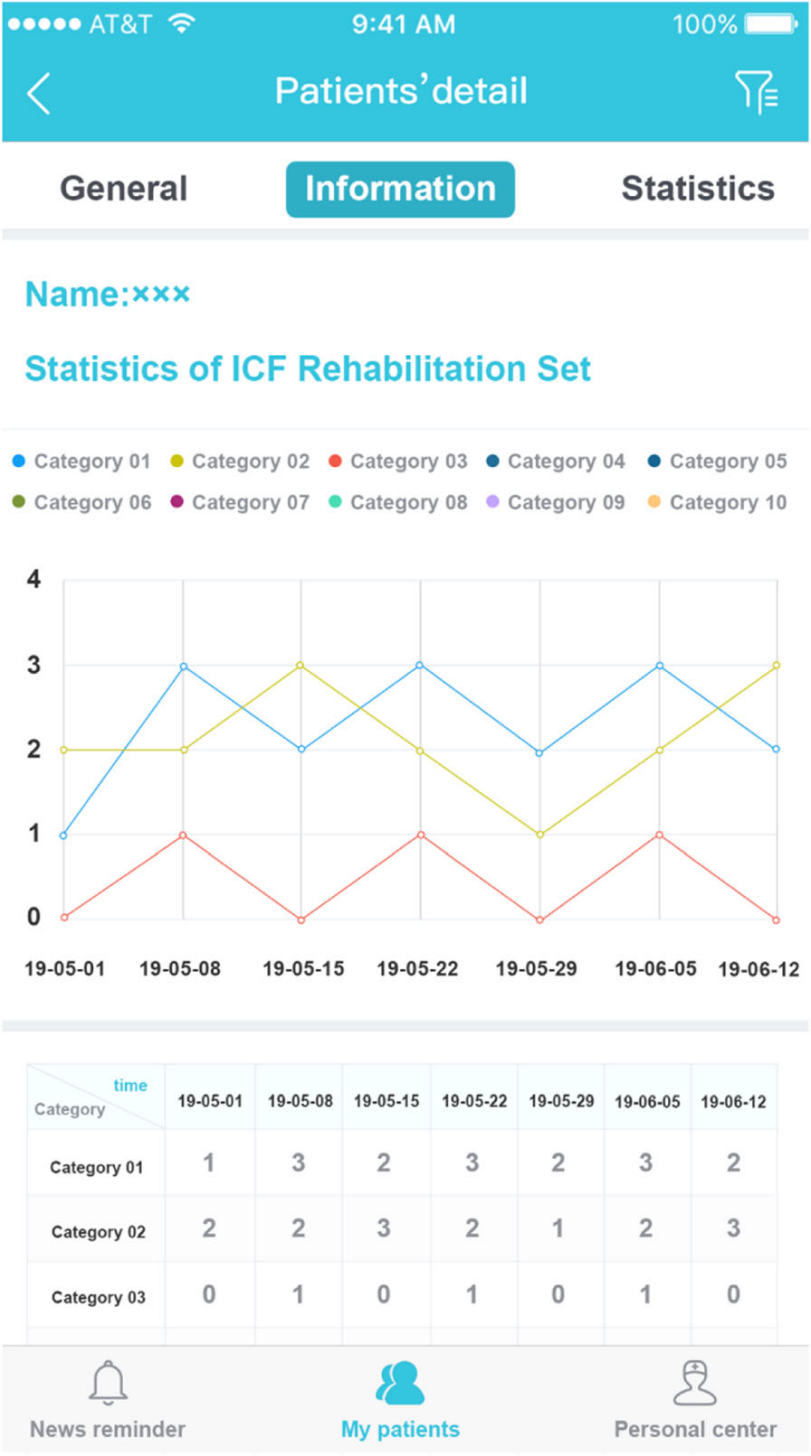
Time trend graph of a patient’ s rating. The time trend graph of the evaluation results about the functional change at different time points for the same patient could be generated automatically

All the patient’ information can be retrieved in the field in the background. Figure 4 presents a sample trend graph of functional change for the same patient at different stages. The data can also be exported to Excel worksheets or printed out.

### Security management

For ensuring data security, password-protected encrypted storage is used. The database includes a white list, and only those on the list can gain access to the data. The security configuration has been set up with cookies and cannot be studied using Java Script. At the app terminal, important local information is stored with md5 encryption and a token which restricts the operation of account information. The key application programming interface uses hypertext transfer protocol over a secure socket layer with post request.

### Pilot study

Five rehabilitation professionals from five cooperating rehabilitation institutions were selected to conduct a 2-week pilot study of the app’s utility. All five had taken part in our previous studies and accepted training on the newly-revised version of the ICF-RS before the pilot testing. All of them had worked in rehabilitation medicine for more than 5 years and had done research on the ICF-RS for more than 3 years.

The participations were sent, through the chat group, an introduction to the app’s development and its purpose. Downloading, installing, and operating the app were explained. The chat group also allowed providing prompt assistance if the participants had any problems during the trial. Each participant assessed the mobile app after using it with at least 5 patients. One-on-one telephone interviews about the user experience were organized to give advice for further app modification. The telephone interviews were recorded and subsequently analyzed to extract the professionals’ candid opinions about the app and its use. The telephone interviews included the following questions:(1) What are your feelings after using the platform? (2) What difficulties did you encounter in using it? (3) What needs to be improved? General comments and suggestions were also solicited.

Besides the five participants, another fifteen professionals who also took part in latest training class for the ICF-RS were used to check the evaluation by team raters. A doctor, a nurse, a physiotherapist, and an occupational therapist from each of the five collaborating institutions were included.

## Results

The app development procedure started in April 2018 and was completed in May 2019. The Agile development was used and the whole process was iterative. It included the developing steps of single evaluation version of ICF-RS, team evaluation version, rehabilitation interventions, and perfecting graphing function. Each iteration took about 8 weeks. The company delivered a revised version of the app at the end of each iteration, and the feedback was provided to the company for some adjustment as required. After passing internal environment test of the app, the technicians registered with the local Terminal to generate the unique key keystroke.jks in the production environment, and further reinforced configuration for the application package, including version number, version name, application package name and other relevant information. Open source code confusion with Android Studio to avoid the third party to decipher and steal source information. Package was released to a third-party application hosting platform and provided download links when it was generated and verified. At the same time, Git management tool was adopted to identify Tag for the specified version number and version update remarks, in order to perform standard management of app deployment. A mobile phone or a tablet computer running version 4.3 or later of the Android operating system is needed. The app was delivered to the end-user by providing website or the quick response code for download.

Five professionals were selected to give advice for trial use: three rehabilitation physicians, a physiotherapist and an occupational therapist. Twenty-five patients were evaluated in the pilot study using single evaluation and another five received team evaluation. The shortest evaluation time was 7 min, and the longest was 28 min. The average evaluation time was 17.4 min. The categorization of the participants and the evaluation times are shown in Table 4. The interface responded quickly and was considered user-friendly. Each module of the app worked as designed. No failures were recorded during the trial, and the data could be retrieved in both the app interface and the background system. The pilot study generated the following typical responses from the five participants.

1. It was very useful that when I filled in the wrong information or the information input was in the wrong format the system could give me a text reminder. But the reminder lasted only a short time so I could not see it clearly.
2. I felt that the whole evaluation process was quite smooth. The main difficulty I encountered was that the admission number and the bed number could only be recorded using numbers. It is a combination of letters and numbers in my hospital. In the evaluation process I found some spelling errors for some ICF categories.
3. The app was easy to use, and I quickly mastered how to operate it. The problem was that I didn’t want to input my ID card number. That infringes my personal privacy. In addition, it was not possible to jump to the next category automatically.
4. I felt that data input with the app was much faster and more convenient compared with the paper version.
5. The evaluation guidance helped me quickly understand the details of the assessment rules and the matters needing attention. It really helped me a lot. One problem was that some sections required multiple choices but the app did not remind me.

**Table 4:** Demographic characteristics, evaluation time of the participants

Name	Profession	Profession title	Working years	Evaluation time for ICF-RS (minute) (mean, max, min)
YX	Physician	Vice senior	17	17 (10, 25)
YZ	Physician	Senior	30	18.4 (11, 26)
WS	Physiotherapists	Attending	10	16.6 (7, 28)
XTZ	Occupational therapists	Attending	10	17.2 (11, 28)
ZLL	Physician	Vice senior	24	17.8 (10, 23)

All the feedback was organized and sent to the software development engineer for further modification and adjustment. The reminder was adjusted from 2 s to 5 s. The display of the telephone number and the ID card number were replaced by asterisks. The spelling errors for some categories were corrected. The admission and bed numbers were modified to allow any combination of letters and numbers. Jumping to the next category automatically during an evaluation was implemented.

## Discussions

The ICF is part of the WHO’s Rehabilitation 2030 initiative [26]. With today’s rapid data accumulation, combining mobile technology with rehabilitation seems to be a development direction for the future [27, 28]. Although the assessment forms based on the core sets and updated information about the ICF can easily be obtained from the WHO ICF website, no details about the operational items are provided. This study sought to provide them. The biggest difficulty during the development process was that the technicians and medical personnel did not understand each other’s expertise. A collaborative group and timely communication were, however, effective for ensuring the project’s progress. The pilot test results show that the development process was eventually successful and each module of the app worked normally. The average evaluation time of the ICF-RS using the app was similar to that with the paper version in a previous study. But when evaluation is complete, the mobile app handles assessment appointment, rehabilitation management and presenting the time trends graphically. That constitutes an example of effective combination of the ICF and networking technology.

Professionals and their patients are the two main users of the mobile health apps reported in previously-published studies. Some apps were developed to promote patients’ self-management, interaction between professionals and patients, disease screening or learning of disease knowledge with patients as the main users [29–32]. Others were developed to cut costs and improve health outcomes with professionals as the main users [33–35]. The app developed in this study belongs to the latter category. Its main advantage is its ability to promote online learning about the evaluation. Today the ICF’s assessment standards can only be promoted in China by organizing myriad training classes across the huge nation. Real familiarity can be promoted only by conducting clinical practice classes nationwide. Health care workers at the grassroots level and in remote rural areas do not have opportunities to learn about and master this technology. The mobile app may help to alleviate this problem [36], [37, 38]. All that required is access to the Internet. The standardized assessment rules can be mastered through on-site study and practice. Professionals unfamiliar with the ICF-RS can view the text, pictures or video guiding the evaluation process. The app also issues reminders for the next steps. In addition, incomplete functional evaluation data cannot be submitted unless it is to be supplemented. The final reports can be generated and checked by the raters at any time. These features can greatly promote the accuracy and completeness of the data tracking each patient.

### Limitation

This study’s pilot test was, of course, only a preliminary implementation. One limitation of the study was that only five professionals took part in the one-on-one interviews. We chose them as they had already taken part in other studies involving the ICF-RS and were familiar with the assessment standards. Larger studies addressing this app’s validity, usefulness and user satisfaction are still needed in further research. In addition, the app as described here might be enhance by adding a prediction module designed to predict the functional level to be reached by a patient in different stages of their rehabilitation.

## Conclusions

A mobile app implementing the ICF-RS was developed successfully. Standardized assessment using the ICF-RS can be realized using the app. At the same time, individualized rehabilitation goals and plans can be formulated based on the evaluation results to achieve multi-professional collaboration in the rehabilitation process.

## Supplementary information

**Additional file 1: Table S1.** The assessment standards of ICF-RS.

**Additional file 2.** The assessment contents of Abbreviated Mental Test.

## Data Availability

The data generated and analyzed during the current study are not publicly available due to restrictions associated with anonymity of participants but are available from the corresponding author on reasonable request. The app is available for free download from https://www.pgyer.com/sunyatsen.

## Abbreviations

app: Application
ICF: International Classification of Functioning, Disability and Health
ICF-RS: International Classification of Functioning, Disability and Health Rehabilitation Set
