# Supplementary material for: A mobile app implementing the international classification of functioning, disability and health rehabilitation set
Source: BMC Med Inform Decis Mak. 2020 Jan 28;20:12. doi: 10.1186/s12911-020-1019-1 (PMC6988202; doi:10.1186/s12911-020-1019-1)
Supplement: Supplementary file 1 — Additional file 1: Table S1. The assessment standards of ICF-RS. [file 12911_2020_1019_MOESM1_ESM.pdf]

Table S1 The Chinese assessment standards of ICF-RS

The category list	The assessment standards of ICF-RS
1.b130 Energy and drive functions	Did you have a lot of energy?<br>a) An extreme amount<br>b) Very much<br>c) A moderate amount<br>d) A little<br>e) Not at all
2.d920 Recreation and leisure	Is your recreation and leisure influenced by your current physical condition?<br>a) No<br>b) Mildly<br>c) Moderately<br>d) Severely<br>e) Completely
3.d850 Remunerative employment	Is your salary be influenced by your current health condition?<br>a) No<br>b) Mildly<br>c) Moderately<br>d) Severely<br>e) Completely
4.d570 looking after one's health	Please pick the one which best represents your ability to look after your health.<br>a) Can completely look after my health independently<br>b) Can mostly look after my health independently<br>c) Need assistance to look after my health<br>d) Need continuous support to look after my health<br>e) Cannot look after my health
5.b134 Sleep functions	Do you have difficulty with sleep?<br>a) 0 points on the NRS<br>b) 1-2 points on the NRS<br>c) 3-4 points on the NRS<br>d) 5-9 points on the NRS<br>e) 10 points on the NRS
6.b640 Sexual functions	Do you have any difficulties in sexual functions (drive, penile erection/vaginal lubrication, ability to reach orgasm, and satisfaction from orgasm)?<br>a) 0 points on the NRS<br>b) 1-2 points on the NRS<br>c) 3-4 points on the NRS<br>d) 5-9 points on the NRS<br>e) 10 points on the NRS
7.b620 Urination functions	Do you have urination problems (e.g. polyuria, urinary retention, urinary incontinence?

	a) Completely<br>b) Mostly<br>c) Moderately<br>d) A little<br>e) Not at all
8.d230 Carry out daily routine	Please pick the one which best represents your ability to carry out your daily routine.<br>a) Can independently manage and complete the daily routine<br>b) Can independently manage and complete the daily routine, but need breaks<br>c) Need assistance to manage and complete the daily routine<br>d) Need continuous support to manage and complete the daily routine<br>e) Cannot manage and complete the daily routine
9.d240 Handing stress and other psychological demands	Please pick the one which best represent your ability to handle stress and control psychological emotions.<br>a) Can completely handle stress and control my emotions<br>b) Can mostly handle stress and control my emotions<br>c) Need assistance to handle stress and control my emotions<br>d) Need continuous support to handle stress and control my emotions<br>e) Cannot handle stress and control my emotions
10.d470 Using transportation	Please pick the one which best represents your ability to use transportation (e.g. car, bus, bicycle, train, ship, plane)<br>a) Can independently use all the above transportation<br>b) Can independently use at least one of the modes of transportation<br>c) Need assistance to use at least one transportation<br>d) Need continuous support to use at least one transportation<br>e) Cannot use transportation
11.d640 Doing house work	Please pick the one which best represents your ability to do housework (e.g. washing, using household appliances, disposing of household garbage, excluding cooking).<br>a) Completing<br>b) Mostly

	c) Moderately<br>d) A little<br>e) Not at all
12.d660 Assisting others	Please pick the one which best represents your ability to assist (e.g. assisting others with self-care, movement, communication, interpersonal relations, nutrition and health maintenance; but excluding remunerative employment).<br>a) Completely<br>b) Mostly<br>c) Moderately<br>d) A little<br>e) Not at all
13.d710 Basic interpersonal interactions	Interviewer's judgment about the basic interpersonal interactions of the interviewee (language, physical contact, responding, facial expression)<br>a) No problem<br>b) Mild problem<br>c) Moderate problem<br>d) Severe problem<br>e) Complete problem
14.d770 Intimate relationships	Do you have difficulty in your spousal/romantic relationship?<br>a) 0 points on the NRS<br>b) 1-2 points on the NRS<br>c) 3-4 points on the NRS<br>d) 5-9 points on the NRS<br>e) 10 points on the NRS
15.b280 Sensation of pain	How would you rate your level of pain?<br>a) 0 points on the NRS<br>b) 1-2 points on the NRS<br>c) 3-4 points on the NRS<br>d) 5-9 points on the NRS<br>e) 10 points on the NRS
16.b455 Exercise tolerance functions	The use of auxiliaries does not affect the score of exercise tolerance.<br>a) The patient is not restricted in his cardiovascular capacity<br>b) The patient is restricted when ascending stairs due to diminished cardio capacity<br>c) The patient is restricted when walking in the corridor due to diminished cardio capacity<br>d) The patient is restricted when walking in a room due to diminished cardio capacity<br>e) The patient is severely restricted due to diminished cardiovascular capacity, so

	that he is only capable of lying.
17.d410 Changing basic body position	Changing body position from (1) lying down (2) squatting (3) kneeling (4) sitting (5) standing (6) bending (7) shifting the body's center of gravity<br>a) Can independently change all the above body positions<br>b) Can independently change 6 of the 7 body positions<br>c) Can independently change 4-5 of the 7 body positions<br>d) Can independently change 1-3 of the 7 body positions<br>e) Cannot independently change body position
18.d415 Maintaining a body position	Maintaining (1) kneeing (2) squatting (3) sitting (4) standing<br>a) Can independent maintain all the body positions above<br>b) Can independently maintain 3 of the 4 body positions<br>c) Can independently 2 of the 4 positions<br>d) Can independently 1 of the 4 body positions<br>e) Cannot independently maintain any of the above body positions
19.d420 Moving around	(1) crawling (2) climbing (3) running (4) jogging (5) jumping (6) swimming<br>a) Can independently moving around by using 4 or more than 4 above ways<br>b) Can independently moving around by using 3 of the 6ways<br>c) Can independently moving around by using 2 of the 6 ways<br>d) Can independently moving around by using 1 of the 6 ways<br>e) Cannot independently moving around
20.b710 Mobility of joint functions	Please tick the box if the patient has restricted mobility in joint (shoulder, elbow, wrist, hand, hip, knee, ankle, foot, cervical vertebra, trunk)<br>a) No tick<br>b)1-4 ticks<br>c) 5-8 ticks<br>d) 9-17 ticks<br>e)18 ticks
21.b730 Muscle power functions	Please tick the box if the patient's muscle power is less than Manual Muscle Testing M4 (shoulder, elbow, wrist, hand, hip, knee, ankle, foot)

	a) No tick<br>b) 1-4 ticks<br>c) 5-8 ticks<br>d) 9-17 ticks<br>e) 18 ticks
22.d510 Wash oneself<br>.	Bathing involves cleaning, rinsing and drying areas from the neck to the feet.<br>a) Self-bathing can be done in any appropriate way without any supervision, prompt or assistance from others<br>b) Except for assistance in preparation and cleaning, you can take a shower; or needs someone to supervise or prompt from the side to ensure safety in the process<br>c) Be able to participate in most activities, but still need assistance from others in less than half of the process to complete the whole activity<br>d) Participate to some extent, but require assistance in half or more of the activities<br>e) Totally rely on others to finish the bath
23.d520 Takes care of all parts of the body	Body care includes washing your face, washing your hands, combing your hair, keeping your mouth clean (including dentures), shaving (for men) and applying makeup (for women in need).<br>a) No need for supervision, tips or assistance. Men can shave their own hair, while women can make up and comb their own hair<br>b) Except for preparation and packing, you can take care of all parts of your body by yourself; or you require supervision or prompting to ensure safety in the process<br>c) Be able to participate in most activities, but still need assistance in less than half of the process<br>d) Participate to some extent, but need others' assistance in the whole process<br>e) Totally rely on others for personal hygiene.
24.d530 Toileting	This includes sitting down and standing up on the lavatory bowl, removing and putting on trousers to prevent soiling clothing and the surrounding environment, using toilet paper and flushing the toilet after use.<br>a) Can use any appropriate method to go to the toilet, without the presence of others supervision, tips or assistance

	b) Except for the need of assistance in preparation and cleaning up, you can go to the toilet by yourself; or you require supervision or prompting to ensure safety in the process<br>c) Be able to participate in most activities, but still need assistance in less than half of the process<br>d) Participate to some extent, but require assistance in half or more of the activities<br>e) Totally rely on others to help go to the toilet.
25.d550 Eating	Use appropriate utensils to transfer food from the container to the mouth. The whole process involves chewing and swallowing.<br>a) Can eat by oneself, without the presence of others to supervise, prompt or assist<br>b) Except for assistance in preparation or packing, the assessed can eat by himself or herself; or you require supervision or prompting to ensure safety in the process;<br>c) Can use tableware, usually a spoon or chopsticks, but still need assistance in less than half of the process;<br>d) Can use tableware to some extent, usually spoons or chopsticks, but require assistance in half or more activities;<br>e) Totally rely on others to help you eat.
26.d540 Wearing	Dressing includes putting on, taking off and fastening clothes; Waist circumference, prosthetics and orthotics are also included when necessary.<br>a) Dress by oneself, without the presence of others to supervise, prompt or assist;<br>b) Except for the need of assistance in preparation and packing, you can dress yourself; or you require supervision or prompting to ensure safety in the process<br>c) Participated in most activities, but still needed the assistance of others to complete the whole activity in less than half of the process<br>d) Participate to some extent, but require assistance in half or more of the activities;<br>5) Completely rely on others to help dress
27.d450 Walking	Walk for ten meters on the flat ground. The patients may wear and remove support or artificial limbs when necessary.<br>a) Walk 10 meters by yourself, without

	other people's supervision, hint or assistance<br>b) Can walk a distance, but cannot complete 10 meters; or need someone to supervise the reminder from the side to ensure safety<br>c) Be able to participate in most walking activities, but still need assistance in less than half of the process to complete the whole activity<br>d) Participate in walking to some extent, but require assistance in half or more of the activities<br>e) No walking at all.
28.d465 Use devices to move around	The participant is required to control the wheelchair and move at least 10 meters including pushing the wheelchair on flat ground, turning and steering the wheelchair to the table, bedside or bathroom.<br>a) Can fully control the wheelchair and move at least 10 meters without the need for other people to supervise, prompt or assist<br>b) Can drive the wheelchair forward, backward, turn and move to the table, bedside or bathroom, etc., but still need assistance in preparation and packing; or the process needs someone to supervise or prompt from the side;<br>c) Be able to participate in most activities, but still need assistance from others in less than half of the process to complete the whole activity<br>d) Can push the wheelchair and move a short distance on the flat ground, but need the assistance of others in half or more activities<br>e) No wheelchair control at all
29. d420 Transferring oneself	It includes movement from one surface to another, such as chair to bed, wheelchair to toilet, etc.<br>a) Can move itself by itself without the need for other people to supervise, prompt or assist it<br>b) Except for assistance in preparation or packing, you can move himself or herself; or the process needs to be supervised or prompted from the side to ensure safety<br>c) Participate in most activities, but still

	need the assistance of others to complete the whole activity in less than half of the process<br>d) Participate to some extent, but require assistance in half or more of the activities<br>e) Completely depend on others or require two persons to assist from the side or to use a moving appliance to assist with the transfer
30. b152 Emotional function	In the past two weeks, please evaluate your ability to generate, control and regulate emotions. (mark at 0 ~ 10)<br>a) 0 points on the NRS<br>b) 1-2 points on the NRS<br>c) 3-4 points on the NRS<br>d) 5-9 points on the NRS<br>e) 10 points on the NRS

NRS: Number rating score
