# Supplementary material for: A mobile app implementing the international classification of functioning, disability and health rehabilitation set
Source: BMC Med Inform Decis Mak. 2020 Jan 28;20:12. doi: 10.1186/s12911-020-1019-1 (PMC6988202; doi:10.1186/s12911-020-1019-1)
Supplement: Supplementary file 2 — Additional file 2. The assessment contents of Abbreviated Mental Test. [file 12911_2020_1019_MOESM2_ESM.pdf]

The assessment contents of Abbreviated Mental Test (AMT)

The details of AMT are as follows.

1. How old are you? (+ / - 5 years of age)

2. What time is it?

* Now please remember “42 Shanghai street” and then I will ask you to repeat it later.

3. What year is this?

4. What is this place?

5. Try to identify any of the two people (doctor, nurse, or other)

6. When is your birthday? (month)

7. What is the date of the Mid-Autumn festival (or Dragon Boat Festival) according to the lunar calendar?

8. Who is the current President or governor of Guangdong province?

9. Try counting down from 20 to 1.

10. Now please repeat the address I asked you to remember.
